# “Children Are Not Children Anymore; They Are a Lost Generation”: Adverse Physical and Mental Health Consequences on Syrian Refugee Children

**DOI:** 10.3390/ijerph17228378

**Published:** 2020-11-12

**Authors:** Niveen Rizkalla, Nour K. Mallat, Rahma Arafa, Suher Adi, Laila Soudi, Steven P. Segal

**Affiliations:** 1 Mack Center on Mental Health & Social Conflict, School of Social Welfare, University of California Berkeley, 120 Haviland Hall, #7400, Berkeley, CA 94720, USA; spsegal@berkeley.edu; 2 School of Public Health, University of California Berkeley, 2121, Berkeley Way #5302, Berkeley, CA 94704, USA; nourmallat@berkeley.edu (N.K.M.); rahma.arafa@berkeley.edu (R.A.); 3 Political Science & Middle Eastern Studies, University of California Berkeley, 210 Barrows Hall #1950, Berkeley, CA 94720, USA; suheraadi@gmail.com; 4 School of Medicine, Stanford University, 291 Campus Drive, Stanford, CA 94305, USA; lsoudi@stanford.edu

**Keywords:** children, Syrian refugees, physical health, mental health, narratives, family processes

## Abstract

This research examines Syrian refugee mothers’ accounts of the physical and mental health of their children being affected by war traumas and displacement challenges. Open-ended audio-recorded interviews were conducted in Arabic with 23 mothers residing in Jordan. Using a narrative approach in the data collection and analysis, five major themes were identified: (1) children were exposed to diverse war traumatic experiences in Syria; (2) the escape journey and refugee camps threatened children’s lives; (3) displacement and family stressors exposed children to poverty, hostility from local peers, educational and recreational challenges, child labor, and domestic violence (these three major themes were considered as trauma related variables); (4) children were not only directly affected physically and mentally by their own traumatic experiences and displacement stressors, but these experiences were mediated and magnified by familial interrelated processes, evidenced in intergenerational transmission of trauma, harsh parenting style, parental control, and parentification; and (5) adverse consequences of both trauma related variables and family processes directly and indirectly traumatized children and adversely impacted their physical and mental health. We examined the themes that emerged from the data in view of three theoretical frameworks and the impact of trauma in the family system on child development. To conclude, humanitarian organizations that provide services and interventions to refugees need to take into account familial processes and not only individual factors affecting refugee children’s physical and mental health. Further implications on policies and trauma research are discussed.

## 1. Introduction

An estimated 2.4 million Syrian children were forcibly displaced due to the ongoing war in Syria [1]. Millions fled to neighboring countries such as Jordan, Turkey, Egypt, and Iraq. Approximately 10,000 children were unaccompanied [2]. War has devastating effects on people, most notably on vulnerable refugee children witnessing acts of violence and being subjected to atrocities, including death, abuse, and torture [3]. The Syrian war exemplifies this impact. Children witness the death of their parents, family members, peers, and other mass killings, as well as experience incarceration and torture [4]. Holding children in captivity is described as “unchilding” them by stripping their innocence and normal childhood [5]. Additionally, Syrian children suffer from hunger, distress, injuries, and physical abuse [6,7] and are the victims of sexual violence and sex trafficking when traded into sex slavery [8].

Syrian children suffer from poor living environments in the host countries [3,9]. In Jordan, 93% of Syrian refugee families live below the poverty line [1] and are unable to afford healthcare expenses [9]. These financial strains aggravate children’s conditions, depriving them of suitable shelter, food, clean water, healthcare, and educational services [3], which also exacerbate their chronic diseases [10] and compromise their health by acute diseases [11].

War-affected Syrian children suffer from significantly high rates of mental illnesses [12], among the most frequent of which are depression, anxiety, and posttraumatic stress disorder (PTSD) [4,12,13]. In Lebanon and Jordan, 45.6% of Syrian children and adolescents developed PTSD [14], while 53% of Syrian refugee adolescents in Jordan experience high insecurity levels, and 82.5% were exposed to more than four lifetime traumas [6]. Syrian refugee children also experience emotional and behavioral regression including high levels of anxiety, bedwetting, nightmares, and panic attacks [4,15,16].

In addition to war and displacement effects, refugee children suffer from difficulties and stressors faced by their uprooted families and parents [17,18]. Three theoretical frameworks attempt to explain some of these difficulties. The first is family systems theory, which claims that family members’ emotional symptoms and physical illnesses can be unconsciously passed from one generation to another [19]. Children develop their coping styles in the context of observing and interacting with their parents. Therefore, parents’ coping styles determine their children’s responses and influence their coping strategies [20]. In Jordan, Syrian refugee adults suffer from high rates of physical and mental health sequelae, including PTSD and somatic symptoms due to war trauma and displacement hardships [7,21,22].

The second theoretical framework is intergenerational transmission of trauma. Parental distress and traumas are often transmitted to their children through this process, whereby the mental health issues of one generation affects the emotional development of subsequent generations [23]. A study examining former prisoners of war and their families found strong intergenerational effects of trauma on children. Fathers’ captivity traumatic exposure and the subsequent secondary traumatic stress experienced by mothers had negatively affected the mental wellbeing of their offspring [24]. Studies conducted on Holocaust survivors showed that parents’ trauma had adversely impacted children via depression and aggressive behaviors [25]. Children of war veterans with PTSD were also prone to develop aggressive and negligent conduct [26]. Similarly, children of refugees with PTSD have frequently suffered from emotional distress and hyperactivity [18,27]

The third theoretical framework is family stress theory, which claims economic pressures and daily struggles affect parenting style and family dynamics. Reuben Hill, the father of family stress theory, developed the ABC-X threefold model: (A) the event, or crisis-precipitating stressor, (B) family resources, which may assist with coping, and (C) family’s subjective definitions/perceptions of the stressor event (the last two depend on the family’s structures and values). All variables interact with each other leading to (X), the likelihood of a crisis in the family. According to Hill, stress jeopardizes the integrity of family life when it disrupts normal family functioning [28]. Financial strains due to displacement challenges encountered by refugee families impair the ability of parents to provide quality caregiving to their children. Refugee children are therefore the victims of domestic violence and abuse as a result of harsh parenting [17,29,30]. There are only a few studies examining Syrian refugee parents’ stressors and economic struggles as having an impact on harsh parenting style and parental control, which negatively affect the psychological wellbeing of their children [14,16,27].

The needs of Syrian refugee children and their families are enormous and concerning, especially given that an end to the conflict is still not foreseen. However, studies that examine both the health and mental health of Syrian refugee children, taking into account family dynamics, interactions, and processes as contributing to child trauma, are scarce. Therefore, a clear evaluation of their condition and its potential long-term consequences is needed. This study examined war traumatic events and displacement challenges experienced by Syrian refugee children via the narration of their mothers, and the impacts of such events on both their physical and mental health. It aims at expanding the understanding of Syrian refugee children’s experiences of the war, escape journey, transition to refugee camps, and subsequently the displacement challenges in the host country of Jordan, taking into account familial processes and their impacts on the physical and mental health sequelae of such uprootedness. It also aims at voicing refugees’ experiences and stories that are frequently unheard. This study provides a unique perspective due to the fact that it does not only consider the individual circumstances of refugee children, but it also includes the impacts of interrelated processes in the family system. Descriptions of children in our study refer to ages 18 years and below. Findings of this study will be utilized to enhance the understanding of Syrian refugee children’s health, and to promote policies, interventions and services that are more responsive to the dire needs of this extremely vulnerable population.

## 2. Materials and Methods

This study belongs to a larger research project about the physical and mental health of Syrian refugees who live in the urban areas of Jordan. The study examines Syrian refugee children’s experiences narrated by their mothers via the narrative approach [31]. Narrative inquiry methods mainly use stories to expand the understanding of the complex human experience, in which people try to make meaning of their live journeys within social, cultural, and historical contexts. Narratives organize events according to sequences of time phases and places to make a narrative meaning of a plot. This study employed the narrative inquiry approach in the data collection phase, and the narrative analysis method in the data analysis phase, taking into account psychological contexts of war and uprootedness and family dynamics [32].

### 2.1. Recruitment

The first author (N.R.) collaborated with multiple humanitarian organizations that provided services to Syrian refugees in Jordan. Initially, the researcher attended staff weekly meetings and explained the study’s purposes and procedures, and asked for their assistance in recruiting participants. Then, staff members approached Syrian refugee women who sought services at their organization and provided information about the study and offered them the opportunity to participate. Refusal rate is unknown to the researchers, since recruitment was solely conducted by staff members. Women who participated in the study shared their experiences with neighbors/relatives, who then sought out the organizations and asked to participate (snowball sampling). Organizations provided a private room for the interviews and hosted the mothers at the organizations’ working hours. Some refugee mothers who agreed to participate encountered barriers to attend the interview at the organizations (work commitments, child care, transportation, etc.) and preferred to be interviewed in public spaces or at their homes. Incentives were not provided to mothers in the recruitment phase; however, when interviews were completed, all participants were provided with gifts that included food baskets and blankets distributed by organizations. When it appeared that additional interviewees were unlikely to add new information, recruitment was stopped [33].

### 2.2. Data Collection

Interviewees (*n* = 23) were Syrian refugee mothers, ≥19 years of age, living in urban areas of Jordan. Interviews were conducted in Arabic from March to June 2014 by the first author (N.R.) who is an Arabic native speaker and has an extensive background in trauma. They were audio-recorded only after obtaining participants’ consent. Interviews lasted between 40 min to 2 h (M = 52.5 min). Participation was voluntary and anonymous, and only pseudonyms of their choice were used during the interview.

In addition to demographic information, the study explored the following questions: What have your children experienced during the war in Syria? What have your children experienced during the escape from Syria to Jordan? What are the challenges your family is currently facing in Jordan? How would you describe your children’s physical and mental health and yours? Do you have any concerns regarding your children’s feelings and behaviors?

The study implemented the narrative research approach, which consisted of gathering data through the collection of participants’ stories based on individual experiences, and chronologically ordering the meaning and impact of those experiences [31]. The first author (N.R.) presented herself as a researcher with a mental health background who came to Jordan to learn about the conflict of the Syrian people. Additionally, and in an effort to minimize the power imbalance and establish an anti-authoritarian relationship between researcher and participants, and in the hopes of developing mutuality, the researcher presented herself as a Palestinian woman, who had also experienced war-trauma, and therefore wished to study the topic more from other women [34]. She encouraged mothers to share their stories in addressing their subjective experiences and cultural and historical contexts (time and place). When encountered with unfamiliar terms in the storytelling, she asked for clarification from participants [35]. The interview process began with mothers’ hesitance, which later on developed into a trusting relationship and openness in exposing their stories. At the end of the interview process, positive questions such as “what dreams do you have for yourself and family?” were asked to help refugee mothers with closure, and in making them feel embraced and hopeful.

### 2.3. Ethics

Only oral consent was required to ensure a safe space for participation. A consent form was provided and read to participants prior to the interview process. All study procedures, approved by the Committee for the Protection of Human Subjects, University of California Berkeley (CPHS, February 2014) were designed to protect the confidentiality of the participant’s disclosures and the security of the data collected. Participants were informed that they could withdraw at any time or refuse to answer any question that caused discomfort. The researcher explained that if self-harm was disclosed at any time during the interview, she would be required to report and refer the case to authorized treating organizations. In cases when the researcher observed that mothers could benefit from interventions, referrals to appropriate organizations were only done after discussion and agreement from participants.

### 2.4. Data Analysis

The interviews were translated and transcribed from Arabic to English by a team of four researchers (R.A., S.A., L.S., and final edits of N.R.) who are bilingual, from a Palestinian or Syrian background, with specialties in public health, mental health, and political science/Middle Eastern studies. An additional researcher joined the team, in the phase of data analysis (N.K.M), with a background in public health.

This study was analyzed according to the narrative approach [31] and considered both the micro and macro levels of the narratives and their contexts [36]. Repetitive themes were detected by each researcher individually. Group meetings resulted in agreement on the major themes and sub-themes that emerged across narratives, and a code book was developed with different colors assigned for each respective theme. Each researcher analyzed the interviews line by line independently. Group meetings then took place to discuss the division of themes for each line in the interviews. The final decision was coded after the analysis was unanimously agreed upon. The tangled process of transcribing, translating, and editing the interviews, followed by data analysis, was slow due to the secondary traumatization experienced by researchers [37], which required cessation from the materials in order to enable working on them at later times.

**Figure 1:**
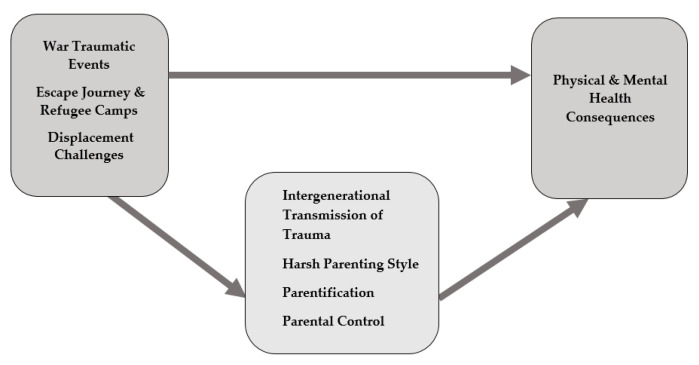
Adverse physical and mental health consequences on Syrian refugee children.

According to the stories of mothers, we constructed our narrative analysis and interpretations of themes and sub-themes, and how family difficulties affected refugee children (inductive process), which were organized chronologically. Additionally, an analysis of narratives was employed (by N.K.M and N.R.), to gain a general understanding of themes evident across the collection of all narratives. Then we found the themes that emerged from the data to be a good fit with three pre-existing theoretical frameworks (deductive process) in examining concepts related to family processes [32]. The narratives were divided into five major themes: war traumatic events, escape journey and refugee camps, displacement challenges in Jordan (all three are trauma-related variables), factors in the family system (mediators), and consequences on refugee children’s physical and mental health. These five major themes were then divided into the following sub-themes (Table 1): 1. War traumatic experiences in Syria: violence and mass killings, hunger and dreadful conditions, physical abuse, separation from family members and loss, incarceration and torture, and sexual violence. 2. Escape journey and refugee camps: escape journey, navigating the refugee camps, difficult weather conditions, decreased quality of food and water, and risk of kidnapping. 3. Displacement challenges in Jordan/family stress: poverty, hostility from local peers, educational and recreational challenges, child labor, and domestic violence. 4. Mediators: Interrelated factors in the family system were also affected by the trauma-related variables, and they indirectly contributed to the adverse impacts on children’s physical and mental health. These factors were identified as mediators: intergenerational transmission of trauma, harsh parenting style, parentification, and parental control. 5. Adverse consequences: trauma-related variables and family interrelated factors negatively impacted children’s physical and mental health (Figure 1).

## 3. Results

### 3.1. Sample and Participant Demographics

The study consisted of 23 Syrian refugee mothers who resided in the following urban areas of Jordan: Amman, Irbid, Ar-Ramtha, Al-Zarqa, and Hiteen. Prior to fleeing Syria, these mothers lived with their children in Aleppo, Homs, Dara’a, Idlib, Sibenyah, Al Moadamyeh, Yarmouk Camp, and Damascus/Aleppo/Dara’a countryside. Table 2 presents participants’ main socio-economic and demographic information. The age of mothers ranged from 21 to 55 years, with the majority being married. Only 4.2% did not know where their husbands were, since they were kidnapped or disappeared. Out of the married women, age of marriage ranged from 14 to 30 years. The majority undertook the escape journey to Jordan with their children and spouses. The number of children in a family ranged from two to eight children. Participants resided in Jordan from 8 months to 5 years, and 30.4% of women were the only parents in the household (fathers were not in Jordan). They lived in one household with two to nine family members. Less than half of households had children attending schools, and 26.1% of mothers arrived to the interview with their children. All the mothers and their children were exposed to traumatic experiences, which impacted at least one child in the family. All names used in the results section are pseudonyms.

### 3.2. Major Narrative Themes and Sub-Themes

#### 3.2.1. War Traumatic Experiences in Syria

Among many concerns voiced by mothers, a primary concern was the safety and wellbeing of their children, hindered by war traumatic experiences. The following are the sub-themes of war traumatic events endured by mothers and their children.

##### Violence and Mass Killings

Children experienced many war atrocities, including shelling and destruction of their neighborhoods and homes, in addition to witnessing violence, mass killings, and arrests, and the death of neighbors, close friends, and relatives. Sana described the fear and horror she and her children felt during the shelling that bombed their neighborhood during the night, “*You know how when a child is sleeping deeply and doesn’t wake up easily… [But when the shelling hit] they stood up just like that on their feet… [Straight up], from the strike that happened. With cries and fear, while I was with them*” (age 29, a mother of three children). Iman recalled “*My children saw dead bodies, they saw people die on the way*”. These sights were imprinted in her oldest child’s memory, which made him still feel terrified, even after finding safety in Jordan. “*If I ask now my oldest child, ‘will you return to Moadamyeh?’ He would answer ‘no, there are snipers and shelling there, I don’t want to’*” (age 22, a mother of three children).

##### Hunger and Dreadful Conditions

Many Syrians needed to endure hunger and other dreadful conditions until they found a path to escape their homeland. Some were internally displaced multiple times and sought shelter in rural regions until reaching safety. During these times, parents were incapable of providing the adequate needs for their children, and therefore children suffered from hunger, cold weather, and malnutrition. Iman narrated, “*There was nothing there [countryside] at all, I stayed there [while pregnant] for two months dying from hunger. There was no food… If they [kids] wanted something, if they craved anything… We could not provide it for them… [it] burnt me from inside a lot*” (age 22, a mother of three children). Sana described the conditions during the winter, “*I lived without gas for around 5 months… I would use matchsticks just to heat the pot to make milk for my son… There was no heater, for two winters… My children did not know the concept of warmth*” (age 29, a mother of three children). Nawal summarized the conditions during the war, “*the children were living like animals*” (age 30, a mother of five children).

##### Physical Abuse

Many Syrian children and adolescents experienced physical abuse and threats to their lives by the Syrian regime. Fatima described an incident her son faced when trying to cross a checkpoint: “*the guard said to [my son],* “*Come here, you! Turn your face to the wall!*” *[My son] told me*, “*Oh, Mama, I swear I was scared. [The guard] kicked me and was going to shoot me*” (age 50, a mother of five children). Yara described a raid in the neighborhood in which her adolescent brother and his neighbor friends were randomly caught when they went out to find what was happening: “*They caught him, hit him with the gun on his head, they folded his hands and took him… They went down on them, on their heads with the guns and their shoes. They would hit them, in a way that was not normal*” (age 21, a mother of two children).

##### Separation from Family Members and Loss

Many children were separated from their natural environment, extended families, and caregivers. Some had lost their loved ones with no anticipation of when they were going to meet them again. Sana described the sudden loss of her husband: “*One day, my husband said goodbye to his kids and went to work… It wasn’t until the end of the night that I got word that my husband was gone. He was kidnapped… It has been two years now*” (age 29, a mother of two children). Shams described her daughter’s continuous difficulty of accepting the separation and loss of her extended family in suggesting creative solutions: “*Mama, let’s go back to Syria… I want to see my aunts, I miss them. Let us go! If we can’t go back to our old house, that’s okay, at least let us go to our grandpa’s new house*” (age 42, a mother of seven children).

##### Incarceration and Torture

Many mothers painfully narrated diverse events in which their children and relatives were incarcerated and tortured. Afaf, whose son was imprisoned for two years by the regime described, “*My son is until now imprisoned and hasn’t been let out… I have been through a lot of agony*” (age 50, a mother of three children). The son has asked his mother not to visit him in prison, due to the severe beating and abuse he would endure before seeing her. Shahd described the incarceration and torture inflicted on a whole family: “*I have a brother in prison; my brother’s children are in prison… From all the torture, he [brother] is now unable to walk on his feet*” (age 36, a mother of two children). Dalal articulated what she witnessed during her work with the regime: “*Atef Najib removed the children’s nails… They took them to jail, they hit them, and they kept them there… They burned them with cigarettes. They skinned one of the boys. They burned them*” (age 48, a mother of two adolescents). Witnessing these atrocities had convinced Dalal to escape with her family to Jordan, leaving behind her senior position with the regime.

##### Sexual Violence

Children experienced sexual violence, whether by being subjected to it themselves or witnessing others. Ghada exposed being group-raped by four soldiers when her oldest son, aged 7 years old was forced to witness the rape. “*They hanged him on the front door and told him ‘watch what we do to your mom’ and swore at him with curses I have never heard before. He was hung there all the time while they raped me… He screamed and screamed all the time and then started crying. I can’t get his cry out of my head until now*” (age 40, a mother of four children). Afaf, who was incarcerated and tortured together with her teenaged daughter, described how she failed in preventing her daughter’s rape, “*I kneeled at the feet of the deputy and told him: ‘I will kiss your feet, take what you want from me but not my daughter, don’t hurt my daughter, I swear she has nothing to do with this.’ She was beaten a lot, subjected to rape, and was humiliated a lot*” (age 50, a mother of three children). Nawal narrated that her 10 year old daughter was subjected to an attempt of rape “*they wanted to rape her, they took off her pants, I swear, we came running and her brothers untied her… if we wouldn’t have reached the house where she was held at, they would have raped her*” (age 30, a mother of five children).

#### 3.2.2. Escape Journey and Refugee Camps

During their escape journey from Syria, children and their parents were exposed to multiple life-threatening risks. However, after crossing the borders and reaching the refugee camps in northern Jordan, new struggles emerged, which were also experienced as traumatizing.

##### Escape Journey

During the escape journey out of Syria, mothers crossed agriculture paths, rural regions, or escaped through the desert and protected their children from many threats. Some fled with their spouses and children, and some undertook the journey by themselves with only their children. For many, the escape journey was illegal, with bribery and smuggling involved, and crossing multiple checkpoints with constant fear of being caught at any time. Feyrouz portrayed this horror journey with her children: “*We walked all the way from Syria to the borders… I had a small baby with me and needed to keep her quiet so no one finds us and knows we are smuggled out of Syria*” (age 36, a mother of seven children). Fatima described how her adolescent son was caught at a checkpoint and was under the threat of being shot at. However, a woman from the crowd, whom her son didn’t know, interfered and advocated for his release: “*She continued to beg the guard to let him go…. My son said that his feet were hitting his head from how hard he was running towards the bus [after being released]*” (age 50, a mother of five children).

##### Navigating the Refugee Camps

After mothers and their children crossed the borders and reached the Zaatari refugee camp in northern Jordan, new challenges in navigating the camp emerged. The camp was unequipped to host the influx of refugees; families were only provided with tents and blankets and at times with mattresses. Facilities in the camp were difficult to reach or unsafe for usage; as Sana narrated, “*There were no toilets. It would take 30 min to take a child somewhere. If you wanted to get water, you would have to carry it from far away to the tent*” (age 29, a mother of three children). Yara articulated her concerns: “*diseases you don’t even know... spread through the babies… If one child got sick, then that’s it, he can’t be healed*” (age 21, a mother of two children). Mothers also disclosed their concerns in regards to safety, violence, and inadequate environments for children as follows, “*The [camp’s gangs] would hit him [son] with rocks, they’d get my kids angry, provoke them, cuss at them… They’d take you if they didn’t steal [from] you first [they would]… They’d throw insects at you… And being catcalled [verbal sexual harassment] with knives… Worse than the mafia*” (Hayat, age 38, a mother of three children).

##### Difficult Weather Conditions

In addition to the challenges in navigating the camp, mothers described intolerable weather conditions both in the winter and summer, which were very different from what they were used to in Syria. The desert climate was new to some mothers who feared for their children’s adjustment: “*the weather was even colder in the night [March], and my daughter was still very young; I just gave birth to her recently*” (Iman, age 22, a mother of three children). Feyrouz was astonished from the weather saying, “*When it snowed, the tent fell over us, we woke up finding the children buried underneath the snow, all wet… And during the summer the dust and dirt were all over the place, you can’t breathe*” (age 36, a mother of seven children).

##### Decreased Quality of Food and Water

Children suffered from hunger and malnutrition in Syria; however, the conditions in the camp did not improve children’s nutrition. Food distribution was complex in the camp, and parents needed to wait long hours in line to get some basic foods. Still, food was scarce as Najat articulated: “*My little girl wasn’t able to get enough food*”, and when the parents provided it, their daughter, “*didn’t want to eat the Zaatari food*” (age 41, a mother of five children). In addition, the water and food quality were poor, which at times resulted in children’s sickness. Nawal described her daughters’ conditions, “*The food, we put it and then you find sand on it, depends on the tent, tons of sand… All of it [the water] was filled with germs and contamination… The girls were poisoned. They got diarrhea, nausea, and fever. All because of the water*” (age 30, a mother of five children). Eventually, children were starving in the camp, “*The children, it’s like they’re living in Africa from fear and hunger*” (Jawahir, age 32, a mother of five children).

##### Risk of Kidnapping

Mothers feared for their children’s safety in the camp and were anxious about them being kidnapped, enslaved into labor, or being subjected to other maltreatment. Most mothers feared for their teenage daughters and kept them away from gangs who sexually harassed them. Shahd, who was anxious about her daughter said, “*I wish that my daughter died in front of my eyes… It’s better than losing her*” (age 36, a mother of two children). Hayat protected her daughter from sexual harassment and exclaimed in anger, “*How would I give her? Is she an object to be given?*” (age 38, a mother of three children). While these mothers only articulated their concern, Jawahir actually lost her son in the camp, “*I looked for him the whole day and I didn’t find him. The second day, I went from the first tent all the way to the last [while being pregnant], until I finally found him…. He was crying for 2 days saying that he wanted his mother*” (age 32, a mother of five children). Jawahir fiercely debated the woman who captured her son and refused to leave without him. Later on, Jawahir discovered that her son was kidnapped for the purpose of gaining profit from selling him to parties operating outside the camp.

#### 3.2.3. Displacement Challenges in Jordan—Family Stress

Families who left the Zaatari camp to reside in urban areas of Jordan faced significant challenges. As displaced families, they encountered housing difficulties, high cost of living, illegality of work, scarce economic resources, poverty, inability to admit their children into schools, and hostility from locals. Family financial strains forced children to work long hours, resulting in various physical ailments, frequent discrimination, and verbal abuse from employers due to their young age and Syrian ethnicity. Some children were fortunate to be admitted into schools; however, others stayed at home with no access to educational or recreational activities. At schools, children suffered from bullying and hostility of local peers, such as name calling and physical altercations. In addition, living conditions made parents and other family members aggressive toward children, which in some cases made them the victims of domestic violence. All these challenges and stressors were also experienced as traumatizing for families and their children.

##### Poverty

Syrian refugees could not legally work in Jordan and therefore faced tremendous economic difficulties and poverty, which prevented parents from providing basic needs to their children, such as food, clothing, health care, education, and school equipment. Iman admitted that her toddler daughter started walking two months ago, but, “*until now I can’t buy her shoes*” (age 22, a mother of three children). These circumstances were experienced as very stressful and agonized mothers. “*This really breaks my heart… That anything they [kids] desire, they can buy. If she [daughter] desires something, if she tells me about something, and I can’t buy it, I suffer*” (Shahd, age 36, a mother of two children). Sana was anxious about her son’s chronic medical condition and her inability to provide the expensive medication he needed regularly. She narrated “*I am anguished that my son’s medication is not being procured. Thoughts. Thoughts about my kids, and about their future*” (age 29, a mother of three children). Poverty has also limited the ability of parent to seek and obtain health care for themselves and their children, which resulted in degradation in health conditions. This impoverished situation has exacerbated the sense of uprootedness and incapacitation for many parents. “*The kids, they need money, they need food, it’s true they need everything—a home. My husband would ask the neighbors, ‘are you able to get us food? Could you get us a kilo of tomatoes? Some blankets?’*” (Layal, age 29, a mother of four children). The inability of parents to provide for their children led many to feel estrangement, lacking a sense of a real home and belonging. Additionally, the majority of mothers described living with other family members, or with their extended families to save the high cost of rental housing. Sharing their housing arrangements with others took off some of the financial burden placed on parents; however, it added other stressors to the family dynamics and increased conflicts and discomfort frequently experienced in crowded housing.

##### Hostility from Local Peers

Many children were not admitted into schools, but the fortunate ones who attended school faced violence by local peers, name calling, and hostility. Ruqayya was helpless and voiced emotionally, “*My boy who is in sixth grade is subjected to beatings at school… And we [parents] tell the teachers, and no one… [protects him]*” (age 39, a mother of seven children). Fatima articulated her concern about violence at school and bullying targeting her son: “*One kid has beaten my son… It got me scared… When they would see him, they would say,* “*Hey Mouaz! [Pseudonym] The Syrian, the Syrian!*” (age 50, a mother of five children). Fatima warned her child to keep silent and not to fight back, out of fear that he would be expelled. Children were not only targeted by local peers at schools but also in their neighborhoods. Rimas was terrified about her son’s safety when he was attacked by local peers while playing in the neighborhood: “*Four boys came and attacked him from behind with a knife on his neck, asking for the ball he had*” (age 48, a mother of six children). This hostility implemented with violence caused anger in one of the mothers as it dehumanized her children: “*We had problems in the neighborhood when kids would beat my kids and tell them ‘Syrians’, there was a period when they didn’t call my kids with their names, [only] ‘Syrians’, and when someone calls you in this way, you perceive it hard. What is it ‘Syrians?’ We are all humans*” (Aya, age 35, a mother of four children). Hostility and violence increased mothers’ fears and anxieties and subsequently their need to protect their children from the outside world and further harm. Children, on the other hand, perceived this hostility as a rejection of their existence, and as being unwanted in Jordan, and thus frequently asked to return home (Syria), or inquired about when their families would be able to return home.

##### Educational and Recreational Challenges

Children who attended schools faced educational challenges due to the curriculum taught in Jordan that differs from the one in Syria, adjustment to the new environment, emotional difficulties, and difficulties with attention, concentration, and memory. All mothers were concerned about the future of their children and their acquisition of education. Some mothers sympathized with their children’s difficulties and tried to rationalize it; as Hayat noted, “*My kids couldn’t handle it… My daughter didn’t understand anything. Maybe the way they taught, I’m assuming [pauses], she didn’t comprehend, or her mental health is poor, and she couldn’t handle it*” (age 38, a mother of three children). Other mothers were amazed by their children’s educational struggles and articulated their frustration “*They don’t concentrate. Before the war, he [son] was a top student... Now his average is zero. There’s zero comprehension. There’s a difference in curriculum... That’s the last thing I expected from my child*” (Latifa, age 35, a mother of six children). In addition to the educational challenges children faced, they had scarce recreational activities available for them. This challenge was even more severe among toddlers and children who did not attend any educational program. Iman admitted in sadness “*Poor thing, there is no place for my son to go out to or go in. I open the door of the house, there is a small balcony, and I let him look outside at the people*” (age 22, a mother of three children). Educational and recreational challenges may impose barriers to normal child development, which may socially isolate children and toddlers from their peers, limit their exposure to child-friendly environments and opportunities to play and explore, as well as developing their social interaction skills.

##### Child Labor

Many children had to work to support their families and therefore dropped out of schools, while others who attended school had to still work in the after school hours. Mothers were worried about their children’s health, state of mind, and exhaustion and felt sorry for their children who had to carry the family’s burden at such an early age. Children worked long hours in garages, restaurants, and other manual and service labor and were underpaid. Ruqayya admitted, “*My son is 14 years old, he works for a guy… When he is off he works in a tire shop. He makes 3 liras [$5] a day. They are good, they help cover some things. And the second boy is in the 6th grade, he is looking for work once school is out, he works at a restaurant for one guy, he makes 2 liras [$3.5] a day… From 3pm till 9pm*” (age 39, a mother of seven children). Feyrouz articulated sadly “*My son is in the 7th grade, if you see what he does, you will feel sorry for him, he works from 8 pm until 8 am in a bakery… He doesn’t go to school because he works all night long*” (age 36, a mother of seven children). Mothers felt ashamed that they needed their children’s economic support. Dalal, who used to be well off, claimed that she used to dine with her children in restaurants in Amman, whereas in the current post-displacement, “*Yesterday, I almost burst from crying [crying]. He’s [son] now working in the same restaurant where he used to eat*” (age 48, a mother of two adolescents). Child labor has limited children’s access to education, or made it impossible due to the long working hours, extreme fatigue, and physical ailments. Mothers worried that lack of education would limit their children in finding future dignified occupational opportunities. For some children, education is compulsory by governmental laws; however, for displaced Syrian refugee children who undertook the responsibility of providing for their families, education became a luxury they could not pursue. Child labor has also increased the risk for children’s exploitation, frequent discrimination, and abuse from employers and workplaces due to their young age and Syrian ethnicity. When children are entrapped and have no choice but to endure work conditions and employers’ maltreatment, they are at increased risk for further harm.

##### Domestic Violence

Parents face many daily stressors post-displacement, and their frustration and aggression are projected towards their children, who become the victims of domestic violence. Children were also targeted by other extended family members with whom children resided. Mothers claimed that their husbands became more aggressive towards their children in Jordan. “*He [husband] comes home and hits the kids a lot, a lot… He [husband] is harsh with him [second son], like all the kids*” (Latifa, age 35, a mother of six children). Fathers were described as being agitated easily “*from anything, from a little kid he gets angry*” and the atmosphere at home becomes tense once the father enters and “*we stay annoyed*” (Shams, age 42, a mother of seven children). Domestic violence targeting children was described by mothers as exceeding the acceptable, as in the following case: “*My husband is the type that has changed a lot, from the mental pressure that is very high in here [Jordan]... Hitting, my husband has started here a lot, hitting, he hits my children a lot. It is not normal*” (Nawal, age 30, a mother of five children). Children were exposed to family members’ threatening conflicts, frustration, and aggression, which at times were projected on them in the form of domestic violence. In order to escape domestic violence at these households, children were required to stay quiet, internalize their self-expression and needs, adapt to the stressful atmosphere at home, and adjust to the new circumstances. Such imposed pressures may disrupt children’s development and put them at increased risk of emotional and behavioral problems, isolation, depression, and anxiety, in addition to being subjected to physical harm and injuries.

#### 3.2.4. Mediators—Interrelated Factors in the Family System

War traumatic events and displacement challenge directly-impacted children’s physical and mental health; however, additional interrelated factors in the family system were also impacted by the traumas and challenges families and children faced, which consequently contributed to the adverse impacts on children’s health. Children suffered from intergenerational transmission of trauma, harsh parenting style, parental control, and changes in the family dynamics and roles, illustrated in parentification. All these mediating factors indirectly influenced the health and mental health of children, as disclosed by the following.

##### Intergenerational Transmission of Trauma

Mothers experienced diverse traumatic events, which they have faced with sadness, agony, and silence. They admitted that their responses affected and were projected onto their children, as demonstrated in the following narration: “*Once they [the kids] see me crying, both of them start crying with me… They hug me and they cry with me*” (Shahd, age 36, a mother of two children). Ghada described her attitude towards her children, which was disrupted due to being raped by the regime soldiers “*sometimes I can’t hug them, I don’t know why but I don’t feel I need to [sad tone]. I feel dirty and not a good mother to them, ever since [the rape]*”. The transmitted trauma’s consequences on her oldest son who witnessed her rape was described as follows “*My child didn’t talk for weeks afterwards [her rape]. He was broken, and he is worried about me. He doesn’t leave me for a second since then. He is like my shadow*” (age 40, a mother of four children).

##### Harsh Parenting Style

War traumatic events and displacement stressors have transformed parents’ behavior towards their children into a harsh parenting style. Najat described her state of mind while in Syria: “*The whole time I was staying there I was crying… If my son moved anything, I’d hit him. I’d get annoyed with my kids [restless tone].*” This mother also described her husband’s harshness with their children post-displacement “*He wouldn’t hit them, but he would throw things next to them, like a phone. He would throw things and break them, like glass… He tells me… To get the kids or shut them up*” (age 41, a mother of five children). Shams claimed “*he [husband] is not happy… He takes it out on his kids*” (age 42, a mother of seven children). It seems that parents who were traumatized by the war, and were extremely challenged by daily stressors, and tried to make ends meet could not contain their own frustration and agitation, or could not tolerate their children’s demands, and thus projected their aggression onto their children. These stressors and lack of parental emotional availability, or even physical availability due to long working hours, were displayed in harsh parenting style.

##### Parentification—Children as Adults

From the moment children escaped with their families, they became protective of their parents and were constantly worried about their safety. Dalal described her oldest son’s reaction while they were escaping from Syria and were caught by the Free Army before crossing the borders: “*My son was strong. He told me to be firm. They shot at us and surrounded us with their cars. We were driving through the desert without any lights—it ended up being the Free Army… He wanted to carry me, my bag, his bag, and his eyes were on me and his dad all the time*” (age 48, a mother of two adolescents). The fear of children for their mothers continued even after crossing the borders and while residing in the refugee camps. Jawahir, who lost her toddler son in the camp, went out to search for him, while her oldest son warned her in a worried tone, “*Don’t go, you could get lost or someone could kidnap you*” (age 32, a mother of five children). Parentification of children was also demonstrated in cases where extreme trauma happened to the mother. Ghada, whose 7 year old son witnessed her rape, was anxious about her wellbeing and experienced separation anxiety, and thus undertook the role of her guardian “*He calls me every 15 min and asks where I am at and what I do, he is always suspicious of new people talking to me, he wants to make sure that nothing happens to me*” (age 40, a mother of four children). Children’s emotional involvement was also witnessed in less traumatizing cases, when children faced displacement challenges as adults, and did not want to overburden their caregiver, as described in the following case, “*They [children] do not take it out on me. They calm me down… They worry about me*” (Karima, age 46, a mother of seven children). Additionally, familial dynamics were changed post-displacement, wherein children undertook a parental role in providing for their families and endured abusive treatment from their employers and harsh working environments. Ruqayya recalled asking her 11 year old son who worked in washing the dishes at a restaurant, “*why do you stay submerged in water in the bitter cold [winter]?*” She was astonished to find how he became suddenly an adult when replying “*he [boss] doesn’t want any employees… from where we are going to get money to spend? Mama, we will be patient…. We will endure*” (age 39, a mother of seven children).

##### Parental Control

Mothers narrated that they felt their children were not safe in Jordan, and therefore they enhanced their parental control and limited their access to the outside world. Hayat articulated her concern over losing her children to misconduct, saying, “*There’s nothing like that [video games for children in the neighborhood] here [Jordan]. Either they’re taking pills, or smoking weed, or beating... I would be losing them. So I’d tell them to stay at home… When they want to leave, I take them out… They can do whatever they want as long as they are in front of my eyes*” (age 36, a mother of seven children). Mothers were also afraid that their children would be subjected to violence while playing in the neighborhood. Aya narrated in a sad tone “*Because of that [violence in the neighborhood], I don’t let them go out much… I tell them, ‘go help your dad’ [works nearby], they go out for one hour or two, or they play here in front of my eyes in the neighborhood’s front yard… An hour and then I get them into the house*” (age 35, a mother of four children). Ruqayya, who was concerned about her children being kidnapped or raped while using public transportation to attend school or work said “*I make my children aware, I tell them if anyone says they know you… Or if they say they are with an organization, ‘come so I can give you some help’, be careful, from home to school and from school to home…. Everything, I am scared of everything... If it was up to me I wouldn’t let them go to school or work*” *[sighs, crying]* (age 39, a mother of seven children). The anxiety and fear for their children forced mothers to adopt parental control strategies. However, as much as these strategies were meant to limit risks and threats to children’s safety, they also limited their freedom and natural development.

#### 3.2.5. Adverse Consequences

All mothers and their children were exposed to war traumatic experiences in Syria, and faced post-displacement challenges and stressors. Mothers narrated that at least one child in their family was adversely impacted by these war events and the new living circumstances in Jordan. These consequences on children’s physical and mental health were also impacted by the interrelated factors in the family system (mediators) described above.

##### Mental Health Consequences

The Syrian war and subsequent displacement challenges took a mental toll on refugee children. Children experienced emotional and behavioral difficulties, as well as developmental regression, including high levels of anxiety, panic attacks, rehashing or enactment of the traumatic events through violent play/fantasy play, depression, aggression, acting out, bedwetting, stuttering, nightmares, sleep disturbances, social boredom and isolation, insecurity, and suicide attempts. Aya cried when narrating how her children started bedwetting in Syria: “*The kids who are old and didn’t pee started peeing… The kids became muted [lost speech] from fear after bombings*” (age 35, a mother of four children). The high levels of anxiety were also described by Najat: “*Planes started to bomb and fire rockets, and my daughter fainted in my lap… From fear [scared tone]*” (age 41, a mother of five children). Ruqayya said that her three year old twin sons would run into the house when seeing the military soldiers screaming in panic, “*Mommy, mommy, they are going to take us.*” However, their anxiety did not decrease while in Jordan: “*once they heard an airplane they would come crying ‘mama, mama the airplane is coming, it is going to hit us, it is coming here to hit us now’… They are small kids*” (age 39, a mother of seven children). Shahd described how witnessing a traumatizing event in Syria has affected her son: “*we had a neighbor who was looking for his son, during gunfire, and then a bullet hit the boy and he fell from the fourth floor to the ground. My son saw him. He was affected by this sight for a long time. He was crying a lot, he still cries sometimes now*” (age 36, a mother of two children). In Jordan, children were also suffering from nightmares and parents found themselves helpless in soothing them, as described in the following case: “*The kids wake up. They say, ‘Mama, he has a gun! Mama, he’s shooting!’ My husband and I would wake up and we don’t know what to do, especially with my son. We’d carry him and tell him that there’s nothing wrong. There’s nothing, no bullets, nothing. The boy then talks to himself*" (Layal, age 29, a mother of four children). Children also experienced behavioral regression post-displacement, as Yara narrated how her son started to stutter: “*My son gets scared from anything. He would say, ‘mama should I say this?’ because he is scared even from normal words… Normal conversations*” (age 21, a mother of two children). Mothers were also concerned about the general poor mental health of their children and the consequences for the future, as one of them narrated “*the children’s mental health, if we continue in this situation, it will become more tired [worse]*” (Iman, age 22, a mother of three children). Some mothers were worried about their children manifesting symptoms of depression. Shams described her 8 year old son’s withdrawal, saying “*You find that his thoughts disband, and his mental health is depleted... He was harmed from this situation, deeply harmed [sighs].*” The child would tell his mother about his wishful thinking: “*Mom, come on, when are we going to go back to Syria? Here our souls are tightened.*” She continued describing her 10 years old son’s sadness in a worried tone: “*you feel as though his pride is broken… Like nothing makes him content at all… He forces himself to act a certain way. He tries to show that he is happy, but he is not happy at all, not at all is he happy*” (age 42, a mother of seven children). The harsh circumstances were also challenging for teenage children. Nawal complained about the behavioral changes of her 14 years old son: “*he started smoking here [Jordan], he doesn’t listen to me. I try to get along with him in gentle ways, but he drives me crazy most days*” (age 30, a mother of five children). Feyrouz described in hopelessness, “*My daughter has taken a knife and tried to cut her veins*” (age 36, a mother of seven children). Afaf articulated her agony from her daughter’s being engrossed in depression and suicidal attempts after being tortured and raped: “*My daughter stayed here in her room for six months, and wouldn’t go* outside, and tried to commit suicide twice” (age 50, a mother of three children).

##### Physical Health Consequences

War traumatizing events have wounded and injured many children, which for some had irreversible physical health consequences. Hayat described the place for wounded children who suffered from the war violence where she used to work: “*I went two, three times and then I couldn’t go there anymore. I saw small children, and there was a 6 year old girl who had her leg cut off from here [groin]*” (age 38, a mother of three children). Afaf, whose daughter was tortured and raped, narrated the impacts on her daughter’s health: “*My daughter had bleeding in her middle ear. Her jaw went to one side, and she dropped 55 pounds... She used to weigh 110 pounds and now she weighs 55 pounds. And till now her appearance looks scary*” (age 50, a mother of three children). In Jordan, the limited access to healthcare impeded the treatment of symptoms among children. Parents’ inability to afford or obtain basic medical necessities, such as medications and adequate treatment, resulted in children’s deteriorated physical health. Shams, whose son was sick, narrated, “*I told them [hospitals’ staff] ‘I am not going to pay for any diagnosis.’ I took myself and left to the pharmacist. I told him [what my son has]. He gave me medication… I told myself, if he gets better, he gets better, if he doesn’t, then I will be forced to pay for the diagnosis*” (age 42, a mother of seven children). Sana, whose son suffered from a chronic disease, said “*he’s four years old and weighs 10 kg… I went to UNHCR [United Nations High Commissioner for Refugees], and they did not agree to pay me. They said that this medication is long-term, that they couldn’t subsidize it, and that it was expensive*” (age 29, a mother of three children). Other mothers tried obtaining medical care for their children through humanitarian organizations; however, they were not content from the services provided, as described in the following case: “*I have a middle son, at the end of every month he gets sick. He has tonsillitis, they get really swollen... But the doctor told me ‘no, he has nothing’ and that this is because there is no specialist in the Crescent*” (Iman, age 22, a mother of three children). Other mothers narrated that housing arrangements caused their children respiratory issues and frequent illnesses, especially in the winter due to the walls’ moisture and mold, and dust seeps during the summer due to unsealed windows and general negligence of landlords’ regular maintenance. Yara described an unusual incident: “*We got a house that was disgusting, they made the place a dumpster... It was full of spiders; then a spider bit my [younger] son and he got an allergic reaction*” (age 21, a mother of two children). In addition, children who were forced to work in manual labor for long hours have also suffered from physical health issues. Fatima, whose son washes dishes and floors in a restaurant, described the consequences: “*You’d say that his hands were like aluminum foil! His legs were blistered. I told him ‘why is it like that?’ He said ‘from the water’. After a while, when he would wear his socks, they’d be bloody [annoyed and frustrated]*” (age 50, a mother of five children).

## 4. Discussion

This study reveals abhorrent injustices and human right violations committed against refugee families and their children. It provides empirical findings that war events, displacement, and family stressors, as well as trauma in the family system have adversely impacted both the physical and mental health of Syrian refugee children [20]. The narration of mothers demonstrates that war events did not solely contribute to the traumatization of children, but that the escape journey, experiences in refugee camps, displacement, and family stressors, as well as dynamics in the family system post-displacement, were also factors interrelated with the traumatization of children. Studies on Syrian refugee children impacted by traumatic events or displacement challenges mainly focus on either the physical health or mental health consequences, but do not provide a holistic view to include multiple factors that contribute to both consequences. Therefore, this study provides a unique examination of children’s conditions, taking into account individual factors and familial processes in the family system. We will address individual and direct factors first, and then discuss the indirect processes in the family system and their impacts on refugee children’s health. We utilize three theories as conceptual frameworks to discuss the descriptions of mothers in the analysis of narratives [32].

### 4.1. War Traumatic Events and Impacts on Physical and Mental Health

Syrian refugee children who endured war events suffered from acute diseases such as severe diarrhea, meningitis, jaundice, tuberculosis, severe malnutrition, violence-related injuries, and anemia [11], as well as high rates of anxiety, depression, and PTSD [4,6,12,13,38]. In our study, children suffered from the armed conflict in Syria, witnessed atrocities, experienced violence and mass killings, hunger and dreadful conditions, and were subjected to physical abuse, incarceration, torture, and sexual violence. During their escape journey and living in refugee camps, they suffered from difficult weather conditions, decreased quality of food and water, were challenged in navigating the camp, and were at risk of being kidnapped. These events caused physical injuries, limb loss, malnutrition, illnesses, and deteriorated health, as well as poor mental health (e.g., panic attacks, horror, nightmares, behavioral regression, and more).

### 4.2. Displacement and Impacts on Physical and Mental Health

Challenging living difficulties in host countries also have negative ramifications on refugee children’s physical and mental health [3,39,40,41,42]. Syrian refugee children who lived in Lebanon and Jordan suffered from infectious diseases such as upper respiratory infections, pharyngitis, tonsillitis, bronchitis, and otitis media [43,44], as well as high rates of PTSD, anxiety, and depression symptoms [6,7,14,15]. Studies also reported on emotional and behavioral issues that Syrian refugee children and adolescents experienced in the host countries such as educational challenges, smoking, socializing with violent local peers, aggression, and identity confusion [4,15,16,45]. In our study, children suffered from poverty, poor living conditions, peers’ hostility, educational and recreational challenges, dropping out of schools, child labor and abuse by employers, and domestic violence, resulting in both physical and mental health issues, e.g., poisoning, malnutrition, illnesses, emotional and behavioral difficulties, and developmental regression, including high levels of anxiety, panic attacks, rehashing or enactment of the traumatic events through violent play/fantasy play, depression, aggression, acting out, bedwetting, stuttering, nightmares and sleep disturbances, social boredom and isolation, insecurity, and suicidal attempts. Access to care imposes diverse barriers on refugees in Jordan, including the high cost of private health care, medications, unemployment, lack of knowledge of available resources provided by organizations, and social stigma pertaining to mental health services. These barriers have deteriorated both the physical and mental health of refugee children and their families [7].

### 4.3. Trauma in the Family System

Though our study has found that Syrian refugee children experienced war traumatic events, displacement, and family stressors while living in Jordan, which affected both their physical and mental health, these ramifications were also impacted by interrelated processes in the family dynamics. These processes were evidenced in intergenerational transmission of trauma, harsh parenting style, parental control, and changes in the family dynamics illustrated in parentification. Based on the family system theory [19], the intergenerational transmission of trauma [23], and the family stress theory [28], parents’ trauma and difficulties in coping with stressors have adversely impacted their children’s physical and mental health, and in adopting similar coping strategies observed, transmitted, and experienced in children’s interactions with their parents [20]. Taking all these variables into account, we claim that if adequate assistance and interventions are not allocated to addressing Syrian refugee children and families’ needs in Jordan, they are doomed to be a lost generation trapped in the cycles of trauma and stress that might exacerbate the process of “unchilding” [5]. This vulnerable refugee population was robbed from their innocence and normal childhood, wherein children were forced to become adults in a blink of an eye, without a secured future in sight. These intergenerational and familial processes may also have adverse impacts on future generations of refugee families and children if not currently addressed appropriately.

Studies found that war traumatic events, displacement, and family stressors might contribute to the process of intergenerational transmission of trauma [18,23,24,46]. This process was found to impact both the physical and mental health of refugee children [7,16,25]. Syrian refugee parents suffer themselves from war traumatic events and diverse daily stressors in the host country, which impact their own physical and mental health [7,21,22], and therefore put their children at risk of developing similar health issues [16,26].

War and uprootedness have been documented as family stressors [28] influencing family dynamics [16,27,40,47]. In our study these dynamics were illustrated in parentification and familial role changes, wherein children adopted parental roles in trying to protect their parents under shelling, during the escape journey out of Syria, or during the transition in refugee camps. In Jordan, they undertook the role of the main providers of their families, dropped out of schools, and became functioning adults who secured the survival of their families. Studies on parentification claim that children with parental roles in their families might suffer from physical and mental health issues [48,49]. Studies on parentification of refugee children suggest that children might suffer from depressive, anxious, and somatic symptoms, as well as overall psychological distress [48,50]. In our study, children’s physical health deteriorated due to child labor and the long working hours and harsh work conditions, and degraded their mental health, as demonstrated by depressive symptoms of keeping silent about their daily challenges, enduring abuse by employers, and in trying to protect their families with constant worrying. These children were no longer children; they became adults. As Shams narrated about her son, “*You feel as though he is older than his age*” (age 42, mother of seven children).

Harsh parenting style was found to stem from both war traumatic events [26,51] and displacement challenges [52,53,54]. Refugee parents suffer from traumatization, adjustment difficulties, uprooting, and marginality, which can impede their parental emotional availability [55]. According to family stress theory [28], parents who are unable to cope with these stressors might project their anger, frustration, and aggression towards their children [16,27,29]. Similar to other studies, when refugee children suffered from a harsh parenting style, presented in this study by domestic violence, they also demonstrated poor physical and mental health [16,29,51,56,57].

Studies on parental control claim that refugee parents might not feel that the host country is a safe environment for raising their children [16,27], or fear that their children might be subjected to violence, bullying, or other maltreatment [14]. Based on the family system theory [19], parents in our study reacted with over-protective behaviors, presented in parental control, in an attempt to protect their children. They confined children to their homes, tried to isolate them from peers’ hostility and negative influences, debated on sending them to schools, and were agonized due to the lack of child friendly recreational activities. Such parental control accompanied with children’s isolation might have multiple impacts on their cognitive, behavioral, emotional, physical, and social development [16,27]. These negative impacts are not only human rights violations, but also encompass public health concerns.

### 4.4. Limitations

Data collection in our study did not include direct testimonies from refugee children but relied on mothers’ narrations in regards to their children’s conditions. Such narrative methodology depends on the skills of narrators and limits the interpretations of Syrian refugee mothers’ narratives to the content and context that were directly meaningful in their experiences [35]. It could be that mothers who sought services at humanitarian organizations adjusted their narratives with the hope of gaining benefits, and therefore increased or decreased the extent of experiences. Our analysis did not include interpretations and insights on the challenges faced by trauma researchers and the risk of secondary traumatization [37]. Finally, the subjective interpretation of researchers, the small sample size, and homogeneity are also additional limitations to our qualitative study. Nevertheless, this study provides a unique and essential input on refugee children’s physical and mental health, taking into account family processes, especially that in Jordan a heterogeneous population of refugee children may face comparable challenges.

## 5. Implications

Services addressing the needs of Syrian refugee children are scarce in Jordan. Humanitarian organizations, funders, and policy makers must consider refugee children’s physical and mental health. Providing affordable medical care, adequate and professional mental health services, as well as educational and recreational services for children are interventions that might ameliorate the negative impacts on the physical and mental health of Syrian refugee children in Jordan. Policies promoting the employment of refugee parents might improve families’ conditions and mitigate familial stressors, as well as reduce the prevalence of child labor. Organizations providing services to refugees are recommended to deliver holistic care, targeting all family members as a system, especially given that in each family at least one child was negatively impacted by war events and displacement challenges. Additionally, interventions need to be provided under a family friendly umbrella-frame to reduce social stigma, especially related to mental health services. Furthermore, studies examining refugee children and traumatized families need to take into consideration familial and generational dynamics, as these interact in interrelated processes that have impacts on each individual member of the family and the family system as a whole, especially among refugee vulnerable populations.

## 6. Conclusions

There is a dire need to meet refugee families’ survival needs, so that children can have a fulfilling childhood, attend schools, play with their peers, and not fall victim to child labor and abuse. The adverse impacts of war and structural violence on refugees must be mitigated for children and their families to lead an enhanced quality of life. The findings presented in our paper provide some information related to the communalities in children’s health trajectories and may be applied in educational and clinical settings to distinguish between causes, diagnosis, and treatments, as well as for future research.

## Figures and Tables

**Table 1:** Themes and sub-themes of mothers’ narratives in the study.

	Trauma-related variables			Mediators	Outcomes
Major themes	1. War traumatic events in Syria	2. Escape journey and refugee camps	3. Displacement challenges in Jordan	4. Interrelated factors in the family system	5. Adverse consequences
Sub-themes	Violence and mass killings	Escape journey	Poverty	Intergenerational transmission of trauma	Physical and mental health consequences
	Hunger and dreadful conditions	Navigating the refugee camps	Hostility from local peers	Harsh parenting style	
	Physical abuse	Difficult weather conditions	Educational and recreational challenges	Parentification - children as adults	
	Separation from family members and loss	Decreased quality of food and water	Child labor	Parental control	
	Incarceration and torture	Risk of kidnapping	Domestic violence		
	Sexual violence				

**Table 2:** Participants’ socio-economic and demographic information (*n* = 23).

Variables		Range	M	SD
Age		21–55	37.62	8.93
Age of marriage		14–30	20.00	4.59
Number of Children		2–8	4.52	1.85
Resided in Jordan (months)		8–60	19.39	13.26
Members in the household		2–9	5.22	2.23
		%		
Marital status:	Married	82.6%		
	Widowed	8.7%		
	Divorced	4.3%		
	Unknown	4.2%		
Escaped to Jordan:	Alone	4.3%		
	Only with children	30.4%		
	Children & spouses	60.9%		
Economic status:	Low	30.4%		
	Very low	69.6%		
Mothers employment:	Unemployed	73.9%		
	Part time	17.4%		
	Full time	8.7%		
Fathers employment:	Unemployed	60.9%		
	Some work	39.1%		
Households with children attending school:	All attended	45%		
	Some attended	35%		
	None attended	20%		
